# High quality AlN epilayers grown on nitrided sapphire by metal organic chemical vapor deposition

**DOI:** 10.1038/srep42747

**Published:** 2017-02-21

**Authors:** Jiaming Wang, Fujun Xu, Chenguang He, Lisheng Zhang, Lin Lu, Xinqiang Wang, Zhixin Qin, Bo Shen

**Affiliations:** 1 State Key Laboratory of Artificial Microstructure and Mesoscopic Physics, School of Physics, Peking University, Beijing 100871, China; 2 Global Energy Interconnection Research Institute, Beijing 102211, China; 3 Anhui Key Laboratory of Detection Technology and Energy Saving Devices, Anhui Polytechnic University, Wuhu 241000, China; 4 Collaboration Innovation Center of Quantum Matter, Beijing 100084, China

## Abstract

Influence of sapphire pretreatment conditions on crystalline quality of AlN epilayers has been investigated by metal organic chemical vapor deposition (MOCVD). Compared to alumination treatment, it is found that appropriate sapphire nitridation significantly straightens the surface atomic terraces and decreases the X-ray diffraction (0002) full width at half maximum (FWHM) to a minimum of 55 arcsec, indicating a great improvement of the tilting feature of the grain structures in the AlN epilayer. More importantly, there is no inversion domains (IDs) found in the AlN epilayers, which clarifies that optimal sapphire nitridation is promising in the growth of high quality AlN. It is deduced that the different interfacial atomic structures caused by various pretreatment conditions influence the orientation of the AlN nucleation layer grains, which eventually determines the tilting features of the AlN epilayers.

AlGaN-based ultraviolet (UV) emitters and detectors have drawn much attention due to a number of applications, e.g., water purification, disinfection of medical tools and UV curing^1^,^2^,^3^. Due to the lack of low-cost bulk AlN substrates, commercial devices are usually fabricated on AlN/sapphire templates grown by metal organic chemical vapor deposition (MOCVD). However, the large lattice and thermal mismatch between AlN and sapphire generally leads to high dislocation density, which acts as nonradiative recombination centers and then seriously restricts device performances^4^. Therefore it is crucial to obtain high crystalline quality AlN epilayers on sapphire substrates. Considerable efforts are devoted to enhancing surface migration of Al adatoms, and techniques such as pulsed atomic layer epitaxy (PALE)^5^, modified migration-enhanced epitaxy (MEE)^6^ and high-temperature (>1300 °C) MOCVD growth^7^ have been adopted. Sapphire nitridation pretreatment originating from the GaN growth^8^,^9^ has also been essayed to improve AlN quality. However, unlike GaN growth on sapphires, where it is well established that nitridation is the key point to obtain high-quality epilayers, sapphire nitridation for AlN growth is still controversial and suggested to prohibit, as formation of inversion domains (IDs) in AlN epilayers and the resulting rough surfaces^10^,^11^ are the biggest obstacles in the procedure.

In this paper, we have studied the impact of sapphire either alumination or nitridation pretreatments on AlN growth by MOCVD. Our results show that compared to the case of alumination, appropriate sapphire nitridation considerably improves the AlN quality, e.g., straightening the surface atomic terraces and decreasing the X-ray diffraction (0002) full width at half maximum (FWHM). More importantly, the aforesaid nitridation condition is demonstrated to effectively avoid the generation of inversion domains (IDs). The surface morphology and orientation of AlN NL grains on sapphires under different pretreatments is further investigated to seek after the mechanism accounting for the improved quality of AlN epilayers on nitrided sapphires.

A series of AlN samples (A-F) were prepared on (0001) sapphire substrates pretreated under different conditions. The pretreatment conditions were shown in Table 1, including sapphire alumination (Sample A), none pretreatment (Sample B) and sapphire nitridation (Sample C-F). Figure 1 shows the typical *in-situ* monitoring curves for AlN growth, where the black and red lines correspond to the growth temperature and optical reflectance curve, respectively. For Samples A-E (all similar to Sample C as shown in Fig. 1(a)), the average reflectance value stays constant at high temperature, indicating that the expectant layer-by-layer growth mode dominates the HT-AlN growth. While for prolonged nitrided Sample F, the damping reflectance in Fig. 1b) suggests rough surface morphology^12^,^13^, which may be caused by island growth of the HT-AlN epilayer.

To further explore the surface morphology of HT-AlN epilayers on sapphires under different pretreatments, AFM images in 3 × 3 μm^2^ scan size of all samples are taken as shown in Fig. 2, where serial number (a)-(f) corresponds to Sample A-F, respectively. Well defined step terraces are observed in Sample A-E, wherein it is noted that terraces on 7 s nitrided sapphire (Sample C) are much straighter than the others. It means that appropriate sapphire nitridation effectively reduces the planar tensions as well as the density of screw threading dislocations, since both of them are demonstrated to be responsible for the terrace meander^10^,^14^. However, when prolonging the sapphire nitridation time to 100 s (Sample E), obvious terrace meander can be observed, suggesting that an optimal sapphire nitridation condition exists, hereon it is 7 s with 2400 sccm NH_3_ at 950 °C. Besides, it is also observed that Sample F features a rough surface morphology delineated by hexagonal faceted nanocolumns similar to the observations in literatures^10^,^15^, which suggests the possible formation of IDs in AlN epilayers on the long-time nitrided sapphire. This three-dimensional (3D) surface morphology is consistent with the damping reflectance as shown in Fig. 1(b).

The crystalline quality is further checked by FWHM values of XRD symmetric (0002) and asymmetric (10-12) ω-scan curves for samples A-F listed in Table 1. Compared to the alumination (Sample A) and none pretreatment (Sample B) cases, proper initial nitridation (Sample C-E) of sapphires dramatically reduces the FWHM values of both (0002) and (10-12) scan curves, suggesting a lower dislocation density in HT-AlN epilayers. It can be found that when prolonging the sapphire nitridation time from 7 s (Sample C) to 100 s (Sample E), the (0002) FWHM values increase, while (10-12) values decreases. The minimum FWHM values of (0002) and (10-12), 55 and 734 arcsec, are obtained for Sample C and E, respectively. Similar variation trend was also reported in GaN epilayers^16^, though less related physical mechanism has been put forward so far. When prolonging the nitridation time to 600 s (Sample F), (0002) FWHM changes little, but (10-12) value increases to 922 arcsec, which is believed to result from the coalescence of 3D nanocolumns with different polarities as shown below.

Taking into account both AFM and XRD results, it can be found that HT-AlN epilayers grown on 7–100 s nitrided sapphires present better crystallographic quality than ones on aluminized or prolonged nitrided sapphires, of which 7 s is identified as the optimal condition for subsequent HT-AlN growth.

In addition, polarity of these AlN samples has also been checked. Wet etching is performed in molten KOH for 4 minutes to verify the existence of IDs in Sample F, as the Al polarity AlN crystals are more inert than N-polarity ones in this process^17^. Figure 3 displays the AFM image of etched Sample F, where 20-nm-deep triangular etch pits appear in the place of 3D nanocolumns. This phenomenon manifests that the fast growing nanocolumns correspond to N-polarity domains, while the surrounding regions are Al-polarity. The same treatments are also carried out for all the other samples but no significant change of surface morphology is observed, suggesting that Al-polarity dominates Sample A-E. This indicates that the formation of IDs is directly dependent on the nitridation degree of sapphire, that is, only excessive sapphire nitridation would result in IDs in HT-AlN epilayers.

Possible mechanisms of sapphire pretreatment have been further investigated. For the case of alumination, it is generally recognized that excess Al atoms from TMAl adhere to the surface of sapphires by weak metallic Al-Al bonds, and the saturated Al atom film should modify the surface energy of the sapphire substrate, and further affects the surface migration of Al and N atoms^13^. While for the case of nitridation, controversy still exists that a thin intermediate phase Al-O-N compound with cubic^18^, rhombohedral^19^ or amorphous^8^ structures as well as a surficial hexagonal AlN layer^20^ has been reported. In any case, the effect of different sapphire pretreatments is directly displayed by the surface morphology of the AlN nucleation layer (NL). Figure 4(a and b) show the AFM images in 1 × 1 μm^2^ scan size of AlN NL layers on 7 s aluminized and 7 s nitrided sapphires, respectively. It is found that dense grains with larger dimension are observed in Fig. 4(a), while there are only isolated slim grains presented in Fig. 4(b).

The orientations of the NL grains under different sapphire pretreatments are further investigated by XRD ω-scans of (0002) peak. For AlN NL on 7 s aluminized sapphire in Fig. 5(a), obvious multi-curve superposition can be observed, therefore two superposed Gaussian equations are adopted to fit the measured curve. Both the two peaks locates near 18.02°, consistent with the expectance for AlN measurement. Compared with the result of bare sapphire in the same measurement range, we further confirm that the two peaks come from AlN NL. Peak 1 has a FWHM value of 335 arcsec, meaning the uniform orientation of grains with 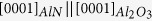, while the FWHM for the stronger Peak 2 is extracted to be 3438 arcsec. Similar FWHM broadening has been reported in ref. 21, where the crystallographic tilt characterized by scanning electron microscopy (SEM) resulted in a significant increase of (0002) FWHM. Researches of grain tilt have been reported in GaN growth on sapphires^22^,^23^, where the GaN plane parallel to the surface of Al_2_O_3_ was confirmed to be (3–302) instead of (0001), and a tilt angle of 19° is observed by transmission electron microscope (TEM). This disorientation was attributed to the lattice mismatch between the epilayers and substrates. Therefore, AlN NL grains on aluminized sapphire will incline even though the mismatch of AlN/Al_2_O_3_ is smaller than that of GaN/Al_2_O_3_, which broadens the FWHM of XRD (0002) ω-scan. Besides, the much stronger intensity of Peak 2 suggests that a mass of grains incline off the sapphire c-axis.

For the 7 s nitridation case, similar two Gaussian peaks are observed in Fig. 5(b), but the intensity ratio of the two peaks changes significantly. The narrow Peak 3 with FWHM of 270 arcsec dominates, while Peak 4 with FWHM of 2905 arcsec is suppressed comparing with the results for alumination case. We conclude that the optimal nitridation condition will effectively relieve the lattice mismatch between AlN NL and sapphire, so that a majority of NL grains have the uniform orientation with 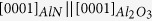.

Based on the above results, physical mechanisms of sapphire alumination and nitridation pretreatments are schematically depicted in Fig. 6. For aluminized sapphires, a saturated Al film adheres on the surface, keeping the internal atomic structures of sapphire unchanged^13^. This Al film would reduce the atomic migration energy on the surface of sapphire, which is beneficial for the formation of NL grains with large dimension. However, this atomic configuration would maintain the lattice mismatch between AlN and Al_2_O_3_, further resulting in the tilted orientation of partial NL grains as shown in Fig. 6(a). Due to the smaller lattice mismatch between AlN and Al_2_O_3_ than that of GaN on Al_2_O_3_, it is reasonable that the disorientation of AlN grains exist but a much smaller tilt angle than GaN case.

While for the nitridation case as shown in Fig. 6(b), the formation of an AlN/AlON composite layer is endorsed from the point of reducing the lattice mismatch between AlN NL and sapphire. When exposing sapphire to ammonia, topmost O atoms have a maximum probability to be substituted by N. The formation of AlN on the surface of sapphire is energetics stable by theoretical calculation^24^ and well-founded in experimental study^20^,^25^, and its thickness depends on NH_3_ flow and nitridation time^20^,^24^,^25^. Beneath AlN, an AlON intermediate layer is proposed as the result of the substitution of O by N in Al_2_O_3_, which has been verified by X-ray photoelectron spectroscopy (XPS)^26^ and transmission electron microscopy (TEM)^19^. The AlN/AlON stepwise structure will effectively relieve the lattice mismatch between AlN NL and sapphire, so that most of NL grains have the uniform orientation with 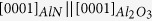 as analyzed in Fig. 5(b). This will lead to much less tilt between different grains during the coalescence process compared to the case of aluminization, which corresponds to the small (0002) FWHM of samples on nitridation sapphires. Moreover, recent research turns out that specific AlON phase is the planar inversion domain boundary (IDB) to change the polarity from N/O to Al, leading to the similar results of Sample C-E in this paper.

The possible generation mechanism of IDs in Sample F is speculated. Excessive sapphire nitridation will greatly increase the substitution of O by N, and then thoroughly change the AlON structure in comparison with the structure proposed for Sample C, leading to the generation of IDs. Similar destruction and disappearance of AlON IDB layer is reported^19^, where it was attributed to the elevated ambient temperatures, the excessive annealing of the buffer, etc. Moreover, when prolonging nitridation time, AlN layer will thicken on the surface of sapphire, forming a barrier for N inward diffusion since N has a lower diffusion coefficient (1.33 × 10^−16^ cm^2^/s) in AlN than in Al_2_O_3_ (8 × 10^−16^ cm^2^/s)^15^. Thus a mass of N atoms adhere on the surface of substrate, building a kind of N-rich condition. Theoretical calculation indicates that IDs can form as Al-polarity and N-polarity structures have very similar formation energies in such N-rich condition^27^.

Based on the optimal sapphire nitridation condition, three alternation cycles of the low- and high-temperature (LT-HT) growth^28^ was adopted to further improve AlN crystal quality. The same growth conditions and film structures as ref. 28. were adopted. It is found that the combination of these two growth techniques can effectively decrease both (0002) and (10-12) FWHM values. Compared to the conventional LT-HT alternation technique (311 acrsec for (0002) FWHM, 548 acrsec for (10-12) FWHM), the introduction of sapphire nitridation pretreatment decreases (0002) and (10-12) FWHM to 130 and 457 arcsec, respectively. Besides, straight atomic steps and a root mean square roughness (RMS) of 0.257 nm (3 × 3 μm^2^) by AFM indicate that the atomically smooth surface of the AlN epilayers can be maintained by combining these two growth techniques.

In summary, influence of sapphire pretreatment conditions on crystalline quality of AlN epilayers has been investigated. It is found that, appropriate sapphire nitridation significantly straightens the surface atomic terraces and decreases the XRD (0002) FWHM to a minimum of 55 arcsec, suggesting the great improvement of the tilting features of grain structures in the AlN epilayers. More importantly, there is no inversion domain found in the AlN epilayers, which clarifies that the method of sapphire nitridation should be promising in the growth of high quality AlN. It is deduced that the different strain state caused by different interfacial atomic structure influences the orientation of the AlN NL grains, which eventually influences the tilting features of AlN epilayers.

## Methods

### Samples Preparation

The samples (A-F) were grown on 2-in. 0.2° off-cut c-sapphire substrates by MOCVD, using an AIXTRON 3 × 2 in. close coupled showerhead (CCS) system. Trimethylaluminum (TMAl) and ammonia (NH3) were used as Al and N precursors, respectively. The growth pressure was maintained to be 85 mbar. Two-step growth procedure was adopted as follows: first, a 20 nm-thick AlN nucleation layer (NL) was deposited on sapphire at 950 °C, and then the chamber temperature was raised to 1240 °C for the growth of 1 μm-thick high temperature AlN (HT-AlN) epilayers. V/III ratio for NL and epilayers was 7500 and 500, respectively. Prior to the NL growth, sapphire substrates were pretreated under different conditions, and the other growth parameters were kept the same for all samples.

### Measurements

LayTec EpiTT was equipped to *in-situ* monitor the reflectance curve (405 nm) as well as the emissivity-corrected surface temperature of the susceptor. The surface morphology were characterized by a Bruker Dimension ICON-PT atomic force microscopy (AFM). The symmetric (0002) and asymmetric (10-12) -scan curves of all samples were measured by a Bruker AXS D8 Discover HRXRD.

## Additional Information

**How to cite this article**: Wang, J. M. *et al*. High quality AlN epilayers grown on nitrided sapphire by metal organic chemical vapor deposition. *Sci. Rep.* **7**, 42747; doi: 10.1038/srep42747 (2017).

**Publisher's note:** Springer Nature remains neutral with regard to jurisdictional claims in published maps and institutional affiliations.

## Figures and Tables

**Figure 1:**
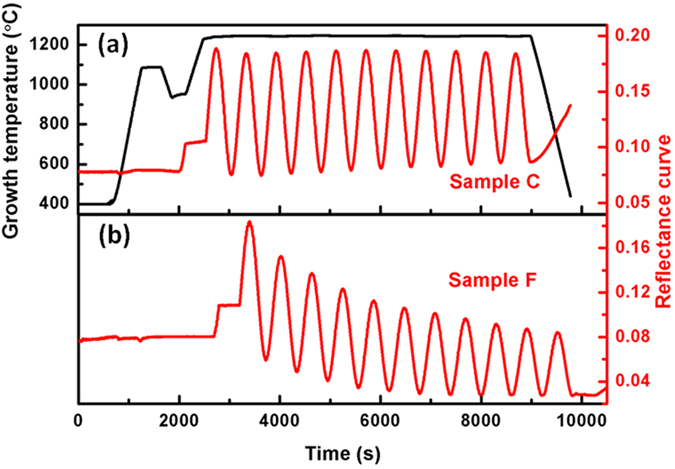
Typical *in-situ* monitoring curves for AlN growth, including temperature (black) and reflectance curves (red), respectively.

**Figure 2:**
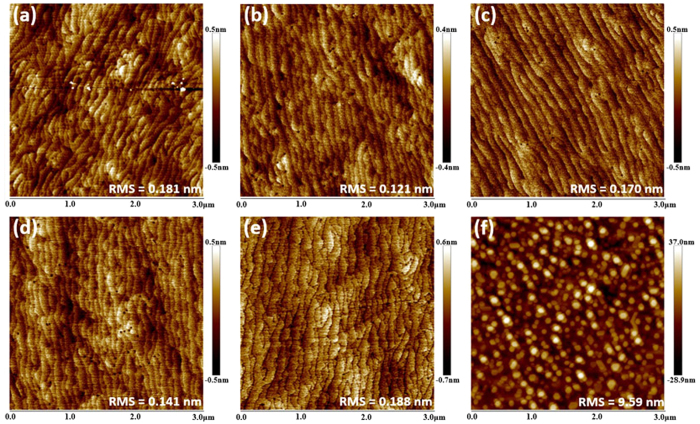
AFM images of the surface morphology for Samples A-F under different pretreatments. The serial number (**a**–**f**) corresponded to Sample A–F.

**Figure 3:**
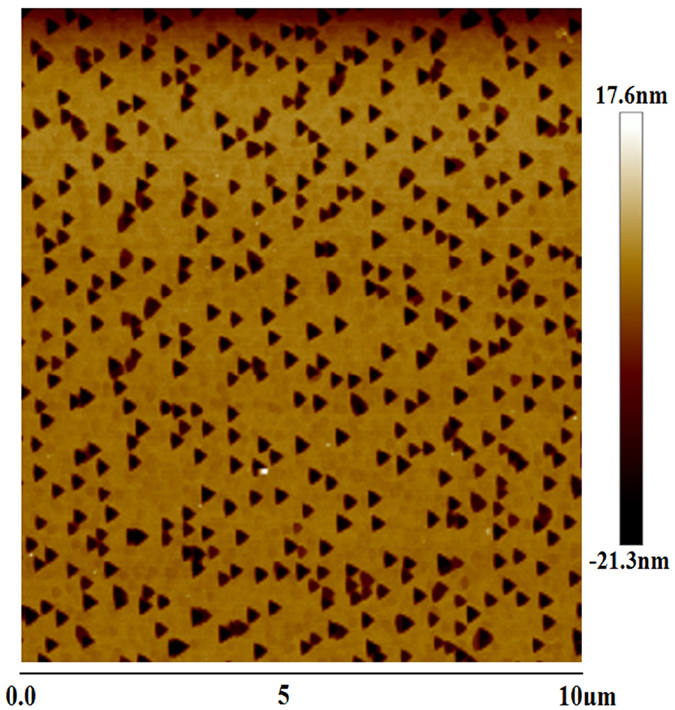
AFM image of Sample F after etching.

**Figure 4:**
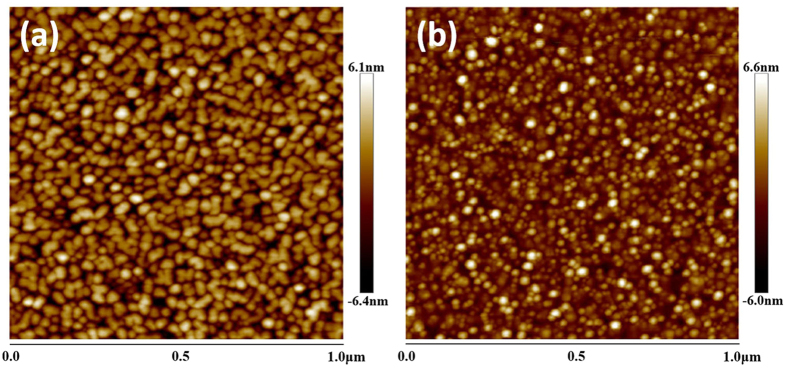
AFM images (1 × 1 μm^2^) of AlN NL on 7 s aluminized (**a**) and 7 s nitrided (**b**) sapphires, respectively.

**Figure 5:**
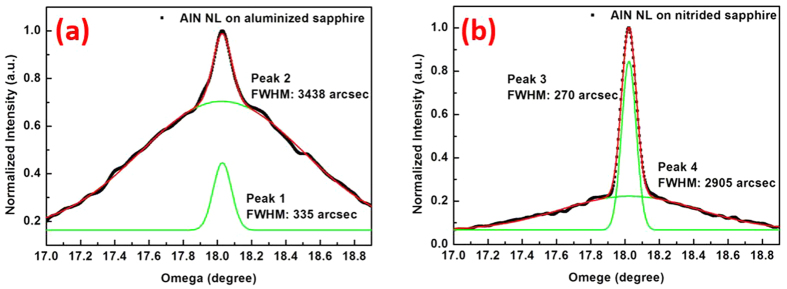
XRD (0002) ω-scan curves for AlN NL on 7 s aluminized (**a**) and 7 s nitrided (**b**) sapphires, respectively.

**Figure 6:**
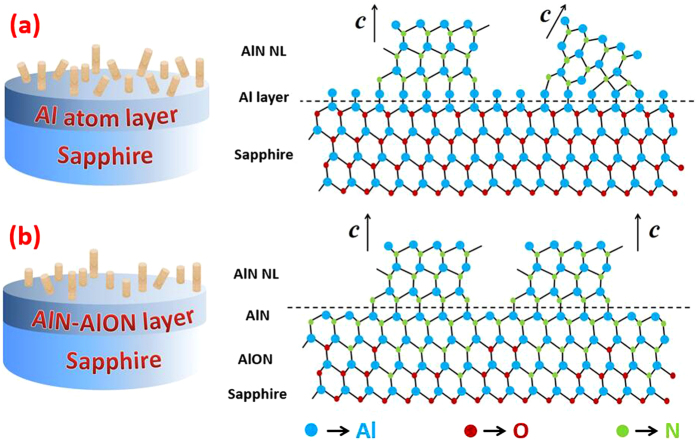
The schematically physical mechanisms of aluminization (**a**) and nitridation (**b**) for sapphire.

**Table 1:** Various pretreatment conditions adopted for sapphire substrates (Sample A-F) prior to growth of AlN nucleation layer, as well as the FWHM values of XRD symmetric (0002) and asymmetric (10-12) ω-scan curves for 1 μm AlN layers following different substrate pretreatments.

No.	Pretreatment	Temperature (°C)	Preflow (sccm)	Time (s)	(0002) FWHM (arcsec)	(10-12) FWHM (arcsec)
A	Alumination	950	40	7	389	911
B	None	950	/	/	81	1019
C	Nitridation	950	2400	7	55	849
D	Nitridation	950	2400	20	93	752
E	Nitridation	950	2400	100	156	734
F	Nitridation	950	2400	600	143	922
